# Supplementary material for: Are carbon emissions trading and green financial instruments synergistic? -Comprehensive quantitative research based on content analysis
Source: PLoS One. 2024 Mar 7;19(3):e0298601. doi: 10.1371/journal.pone.0298601 (PMC10919601; doi:10.1371/journal.pone.0298601)
Supplement: S1 Data — (ZIP) [file pone.0298601.s001.zip › Data/地级市Z_F工作报告.zip!/┴╔─■/┤≤┴1⁄4╩╨/└┤╫╘╣1⁄2╓┌║┼í╛┬φ┐╦╩2╛▌═°í┐_2018.docx]

各位代表：

　　现在，我代表市政府向大会报告工作，请予审议，并请市政协委员提出意见。

　　一、2017年工作回顾

　　过去的一年，在省委、省政府和市委的坚强领导下，我们深入学习贯彻习近平新时代中国特色社会主义思想和党的十九大精神，全面落实新发展理念和“四个着力”“三个推进”，坚持稳中求进工作总基调，以提高发展质量和效益为中心，以深化供给侧结构性改革为主线，以振兴实体经济为重点，扎实做好稳增长、促改革、调结构、惠民生、防风险各项工作，圆满完成市十五届人大六次会议确定的目标任务。

　　一年来，经过广大干部群众的奋力拼搏，有七项重大工作成果令人鼓舞。

　　一是经济转型发展成效显著。面对严峻的经济形势，我们保持定力、沉着应对，采取系列政策举措，实现了经济连续11个季度稳步增长，高于全国平均增速。发展动能加快转换，经济结构优化升级，三次产业比重由五年前的6.4：52：41.6优化为6.4：41.5：52.1，经济增长的持续性、稳定性、协调性进一步增强。

　　二是再次蝉联全国文明城市。在中央文明委组织的第五次全国文明城市考核评比中，我市各项指标排列在前，再次获得全国文明城市光荣称号，成为全国仅有的两个连获五届殊荣的城市之一。

　　三是自由贸易试验区正式挂牌运行。我们坚持大胆试、大胆闯、自主改，复制推广上海等自贸区经验116项，7项创新成果成为全国最佳案例。建立了负面清单制度，实行了国际贸易“单一窗口”，投资便利化、贸易自由化、金融国际化、管理现代化加速推进，成为对外开放的新高地。

　　四是“放管服”改革实现新突破。全市取消调整行政审批事项1760项、精简46.6%，大力提高审批效率，承诺办结时限压缩至6.6天，各类申请材料压缩至4.9件，实现市级网上审批事项达70%以上。

　　五是以“大连光源”为代表的一批科技创新公共平台投入使用。大连化物所大型自由电子激光科学研究装置——“大连光源”建成运行，中国火箭技术研究院、中科院科教融合基地等50家国家级科技创新公共平台成为我市创新驱动发展的重要基地。

　　六是蓝天保卫战首战告捷。广泛动员、全民参战，以最坚定的决心、最大的工作力度、最有效的举措，强力推进生态文明建设，全面打响蓝天保卫战。全年PM2.5、PM10同比分别下降13%和12%，实现了300个蓝天的奋斗目标。

　　七是北水南调入连工程全面竣工通水。总投资49亿元、全长222公里的大伙房水库输水入连工程至2017年11月20日全面竣工，实现了满负荷输水，日输水量达90万立方米，解决了城市3/5的用水量，有力保障了我市对水的需求。

　　以上七项重大成果，事关大连振兴发展，事关700万大连人民切身利益，事关城市核心竞争力，为大连高水平全面建成小康社会、率先实现全面振兴夯实了基础、增添了动力!

　　一年来，我们主要做了以下工作。

　　（一）坚持把稳增长作为首要任务，稳中向好的态势日益巩固。

　　主要指标增势良好。实现地区生产总值7363.9亿元，增长7.1%；一般公共预算收入657.7亿元，增长7.5%；固定资产投资1652.8亿元，增长15.1%；社会消费品零售总额3722.5亿元，增长9.2%；居民消费价格涨幅2.1%；城乡居民人均可支配收入40587元和16865元，分别增长6.7%和7.7%，万元GDP能耗下降3.5%。

　　投资结构日益优化。发挥有效投资对稳增长的关键作用，工业、高技术产业投资分别增长58.6%、94.1%，民间投资增长22.4%。注重大项目带动，开复工亿元以上项目621个，英特尔、恒力项目年内投资分别达到178亿元和200亿元。强化财政资金杠杆作用，推进PPP项目17个，撬动社会投资833亿元。

　　招商引资成效明显。坚持内资与外资并重、引资与引智引技并举，全年组织境内外招商13次，引进省外内资918.1亿元、增长46.1%，实际利用外资32.5亿美元、增长8.2%。夏季达沃斯论坛期间签约重大项目25个，投资额达284.6亿元。

　　消费动能持续增强。开展消费升级促进行动，信息、家居、汽车、旅游、教育、养老等消费旺盛，网络消费成为热点，大商集团销售额超过2800亿元。商品房销售面积、销售额分别增长18.7％和30.9％。全年游客突破8000万人次，旅游业总收入1280亿元、增长12.8%。我市成为“中国邮轮旅游发展实验区”“中国旅游休闲示范城市”。

　　外贸实现稳步增长。着力培育外贸主体、拓展新兴市场、创新业态品牌，加快外贸出口孵化基地建设，新增跨境电商线上平台24家、外贸企业2300家。外贸进出口总额4132.2亿元、增长21.7%。

　　民营经济健康发展。出台支持民营经济发展32条措施，着力帮助企业破解发展和运行瓶颈难题。深入实施“大做强”“小升规”“个转企”工程，实现2000余家存量企业“升规做强”。服务中小企业的“育龙基金”规模达到43亿元，帮助中小微企业争取信贷授信100亿元、融资157亿元。

　　（二）深化供给侧结构性改革，发展质量显著提升。

　　“三去一降一补”深入推进。扎实有效去产能，平板玻璃、粗钢、普钢产量分别下降30%、17.6%和10.2%。因地施策去库存，商品房待售面积下降23.2%，去化周期降至15个月。积极稳妥去杠杆，规模以上工业企业资产负债率下降1.1个百分点。开展降本增效专项行动，累计为企业减免各种税费577.7亿元。实施补短板工程，生态环保投资增长近3倍。

　　战略性新兴产业持续壮大。制定实施新兴产业发展计划，高技术产业和战略性新兴产业增加值分别增长50.8%、17.5%。英特尔非易失性存储器、芯冠科技外延片等一批项目建成，首枚国产轨道交通网络控制芯片通过测试。储能产业稳步壮大，调峰电站示范项目加快推进，储能装备一期工程建成投产。新一代信息技术产业快速发展，贸易大数据平台为全球249个国家和地区提供服务，云计算“大连模式”在全国推广。

　　传统产业加快转型升级。深入实施《中国制造2025大连行动计划》，加速制造业与信息业融合发展，不断提升产品质量和效益。全市规模以上工业增加值增长11.2%，装备制造业增加值增长21.1%。首艘国产航母在大连下水，高端船舶及海工钻采装备形成了规模化、系列化生产能力。瓦房店轴承产业集群提质增效，恒力炼化一体化项目加快推进，省级精细化工共性技术创新平台投入使用，首批12个中试项目入驻运营。汽车制造业、电子信息产业增加值分别增长28.5%和74.3%。冰山集团、惜能科技、捷程伟业分别获批国家级服务型制造业示范企业、示范项目、示范平台。深化质量提升和品牌创建，我市成为全国质量强市示范城市。

　　现代服务业加速发展。港航物流业提质增效，实现海港货物吞吐量4.55亿吨、集装箱吞吐量970.7万标箱，空港旅客吞吐量1750万人次，物流业增加值占地区生产总值比重达到10.6%。市场体系不断完善，新增世界500强和行业百强商贸企业10家。金融服务实体经济的能力进一步增强，新设金融及融资机构29家，新增“新三板”“四板”挂牌企业174家。大商所成功上市豆粕期权，向国际综合性衍生品市场迈出重大步伐。金融业增加值占地区生产总值比重达9.3%。

　　都市型现代农业成效显著。全面完成农村土地确权登记，农业共营制改革试点成效良好，组建农村土地股份合作社25家，入社农户3600户，完成土地流转面积147万亩。转型升级农业产业化龙头企业30家，全市蔬菜、水果、畜禽和水产品平均抽检合格率位居全国前列。推进“互联网+现代农业”，发展农村电商店铺800家。新建国家级海洋牧场示范区4个，发展海洋牧场10万亩，长海县海洋经济加快向现代高质量海洋牧场转型升级，普兰店、庄河分别获评“中国辽参产业之都”和“中国贝类产业之都”。大连都市现代农业综合发展指数居全国第四位。

　　（三）着力推进改革开放，发展活力有效释放。

　　营商环境建设扎实推进。开展营商环境建设年活动，明确十大重点任务，开展专项整治行动，优化六大环境，全力打造亲清政商关系。推进多规合一、多图联审、区域评估、行政审批告知承诺制、多证合一等系列改革，审批事项精简26.9%，申请材料精简42.6%。构建市场主体自律、行业自治、社会监督、政府监管“四位一体”综合监管格局。推进“法治大连”“诚信大连”建设。全年新登记市场主体114288户、增长19.2%；新登记各类企业38728户、增长8.7%，平均每个工作日新增156户。

　　国有企业改革取得新进展。整合优化国有资源，组建房屋经营管理公司和文化产业集团，整合重工•起重与大橡塑、装备投集团与国投集团。加大混合所有制改革力度，金重集团、染化集团混改工作有序推进，东北特钢、燃气集团完成重整改制。全市出资企业资产总额增长3.5%、净资产增长6%。

　　开放型经济新体制加快建立。海关、检验检疫、边防、海事等部门密切配合，在贸易、投资、口岸便利化等方面先行先试，推出一批制度创新成果。深化沿海经济带开发开放，19个重点园区对经济增长贡献率达50%。金普新区、高新区、长兴岛、花园口、旅顺开发区等配置资源要素能力和效率持续提升，产业集聚功能明显增强。参与“一带一路”建设实现新突破，“辽满欧”班列常态化运行，开通国内首条直达斯洛伐克的中欧班列。全年海铁联运完成41.2万标箱，位居全国之首。

　　沪连对口合作取得实效。全面建立沪连对口合作机制，加强高层互访，制定年度合作计划，23项重点合作项目有序推进。加强科技合作，与上海共建国家技术转移大连分中心，实施了13个对口科技合作项目。推进产业对接，集中签约重大产业合作项目57个、投资总额1704亿元。

　　（四）深入实施创新驱动战略，发展方式加快转变。

　　政策体系不断完善。制定科技创新鼓励政策。深化制度创新，在科技管理体制、科技金融结合、创新创业服务体系建设等领域形成一批可复制可推广的经验，中关村“6＋4”创新政策落地实施。

　　创新能力显著提升。发挥企业科技创新主体作用，新增10个产业技术创新联盟、61个创新研发平台，软银中国区总部、IBM创新中心等10个创新项目落户。加强人才队伍建设，引进产业发展紧缺人才343人、海外优秀专家1041人，国家、省百千万人才工程入选261人，新增3名两院院士，在连两院院士达到30人。万人发明专利拥有量16件，我市有14个项目荣获2017年度国家科技奖励。

　　“双创”活力持续增强。积极推动各类创新创业平台建设，同济大学创业谷、弘信创业工场等知名平台相继落户，全年备案众创空间15家，认定科技企业孵化器6家，在孵企业达到1500家，创业团队3000个。高新区、大连化物所入选“国家双创示范基地”。我市“双创”工作得到中央领导的肯定。

　　（五）加强城市规划建设管理，功能品质明显改善。

　　基础设施建设持续推进。启动新一轮城市总体规划编制工作。完善道路交通体系，疏港路拓宽改造、西部通道改建工程顺利竣工。渤海大道即将竣工通车，金普城际铁路基础工程全线贯通，旅顺中部通道、南部滨海大道延伸线等工程加快推进，地铁5号线、大连湾海底隧道及光明路延伸工程、大连北站综合交通枢纽开工建设。维修城市道路100条，新建通屯油路800公里，改造普通公路352公里，新增停车泊位6.2万个。

　　城乡生态环境显著改善。持续开展市容环境综合整治，对占道经营、无证营运、露天烧烤、街巷烧纸、乱搭乱建、乱停乱放等沉疴陋习零容忍。中心城区环境品质和文明程度进一步提升。改造后的小付家庄公园、星海公园向市民开放，植物园改造工程加快推进，椒金山等5座城市山体公园建成使用，甘井子废弃矿山环境整治取得成效。加大生态建设力度，植树1129万株，更新造林8.5万亩，林木绿化率达50%，通过了国家森林城市复查。坚持铁腕治污，实施遏制扬尘污染“五化”行动，取缔燃煤小锅炉950台，淘汰黄标车及老旧车辆2.6万辆，全面禁止秸秆焚烧。严格落实河长制，主城区6条黑臭水体全面治理，7个国考河流断面水质全部达标，跻身全国水生态文明城市之列。市区公共机构生活垃圾强制分类全面启动。认真整改落实中央环保督察和国家海洋督察提出的问题，各项生态指标明显好转。我市获评“2017美丽山水城市”。

　　城市文化品质不断提升。开展精神文明“5+1”创建活动，城市文明程度和市民文明素质显著提升。完善公共文化服务体系，惠民公益演出1600多场，一批文艺作品获奖。加快文化产业集聚区建设，华录集团进入全国文化企业30强。成功举办国际马拉松等品牌赛事，大连女足勇夺全国“双冠王”、男足重返中超联赛。

　　（六）大力保障和改善民生，人民福祉持续增进。

　　民生投入不断增加。全年投入各项民生资金679.3亿元，占财政总支出73.8%。15项重点民生工程务实暖心，完成“暖房子”工程232万平方米，对294万平方米的老旧住宅下水管网进行维修改造，一批群众期待解决的问题得到妥善解决。

　　创业有力带动就业。出台实施一系列促进就业创业政策，建立市区联动就业创业服务机制，本地生源高校毕业生就业率达99.7%，全年城镇新增就业10万人，城镇登记失业率为2.4%。

　　社保体系更加完善。持续加强社会保障，企业退休人员月人均养老金增长到2572元，城乡低保标准提高到每人每月670元和450元，城镇居民医保财政补助标准提高到550元，新农合人均筹资标准提高到700元。加强困难群众帮扶，全面上调特困人员供养、残疾人生活补贴等标准，为7.8万残疾人发放生活和护理补贴。加大精准扶贫力度，实施扶贫项目298个，建成93个，总投资近10亿元，128个低收入村农民人均可支配收入增长18%。

社会事业全面发展。全面提升社会事业普惠化、均等化水平。全市新增公办幼儿园16所、学位3630个，新认定普惠性民办幼儿园59所；新增义务教育集团8个，实现国家义务教育发展基本均衡县全覆盖；大连职业教育基地投入使用；大连理工大学、海事大学分别入选一流大学、一流学科建设高校。全面启动城市公立医院综合改革，58所城市公立医院全部取消药品加成；组建各类医疗联合体29个。复制推广中山区社区居家养老服务“林海模式”80多家，西岗区获批全国首批健康促进区，沙河口区成为全国智慧健康养老示范基地。

　　社会治理体系逐步完善。巩固拓展“大连经验”，深入推进立体化社会治安防控体系建设，实现网格化服务管理全覆盖，我市第六次被评为“全国社会治安综合治理优秀市”。强化安全发展理念，大力推进遏制重特大事故示范城市建设，全市生产安全事故起数和死亡人数同比下降23.4%和13.9%。实施社会矛盾纠纷排查化解，开展领导包案化解信访突出问题攻坚行动，大量信访矛盾妥善解决。组织系列食品药品专项整治和稽查行动，国家食品安全示范城市创建取得显著成效。社区治理服务体系不断完善，92%以上社区达到全国和谐社区建设标准。

　　民族、宗教、外事、侨务、对台、人防、档案、气象、仲裁、防震减灾、双拥共建、民兵预备役和边海防等各项工作均取得新成绩。

　　（七）落实全面从严治党责任，政府自身建设不断加强。

　　把思想政治建设放在首位。坚持把学习贯彻习近平新时代中国特色社会主义思想和党的十九大精神作为首要政治任务，坚持理论联系实际，用创新理论成果武装头脑、指导实践、推动工作。牢固树立“四个意识”，坚定维护以习近平同志为核心的党中央的权威和集中统一领导。扎实推进“两学一做”学习教育常态化制度化，振奋精神、明确方向、履职尽责。

　　依法行政全面推进。强化重大行政决策合法性审查，完善政府权责清单制度，全面推进行政决策、执行、管理、服务、结果“五公开”。自觉接受人大法律监督和工作监督、政协民主监督、社会舆论监督，认真听取民主党派、工商联、无党派人士和人民团体意见。积极回应社会关切，人大代表意见、建议和政协委员提案全部办结。

　　作风建设持续加强。深入开展解放思想大学习大讨论活动，围绕“九个聚焦”，对标上海、凝聚共识。坚定不移落实中央八项规定精神，坚决防止“四风”反弹回潮。大力弘扬马上就办、办就办好工作作风，深入开展破坏营商环境问题专项整治，认真解决少数干部庸政懒政怠政等突出问题，政府执行力和行政效能进一步增强。

　　反腐倡廉力度加大。认真执行《准则》《条例》，自觉践行政府系统“五项承诺”，深入开展中央和省委巡视反馈意见整改，坚决肃清薄熙来流毒和王珉等人恶劣影响。加大审计监督力度，完善政府预算制度，严格“三公”经费预算管理。强化督查督办、考核问责和正向激励，确保党中央、国务院和省委、省政府及市委决策部署不折不扣落实到位。

　　二、过去五年主要工作成效

　　过去的五年，面对复杂多变的外部环境，面对巨大的经济下行压力，面对净化和修复政治生态的严峻考验，我们全面贯彻落实党的十八大及十八届历次全会精神和党中央、国务院关于老工业基地全面振兴的战略部署，按照习近平总书记对大连提出的“两先区”目标要求，认真贯彻创新、协调、绿色、开放、共享发展理念，以坚定的信心、为民的情怀和强烈的责任担当，开拓进取，攻坚克难，推动经济社会迈入持续健康发展轨道，在全省较好地发挥了振兴发展的排头兵和领头羊作用。

　　五年来，我们坚定不移推进创新发展，新旧动能转换实现新突破。实施创新驱动发展战略，科技创新能力大幅提升，一批具有自主知识产权的关键技术或核心部件，助力华龙一号、中国天眼、982钻井平台等大国重器建成。大众创业、万众创新蓬勃开展，建成国家级创新平台50个、省级创新平台393个、市级创新平台386个。我市获批国家自主创新示范区和知识产权示范城市。

　　五年来，我们坚定不移推进协调发展，结构优化升级形成新格局。产业结构调整取得可喜成效，高技术产业和装备制造业增加值占规模以上工业增加值比重分别由5.5%、44%提高到17.3%、47.8%，服务业占GDP比重由41.6%提高到52.1%，经济增长实现从主要依赖投资和出口支撑转向更多依靠消费拉动、创新驱动。全域城市化加快推进，“四大组团”协调发展，普兰店撤市建区，全市城镇化率提高到78.3%。城乡路网、电网、水网逐步完善，国际会议中心、体育中心、南部滨海大道等一批重大工程投入使用，地铁1号、2号线建成并投入运营。

　　五年来，我们坚定不移推进绿色发展，“美丽大连”建设迈出新步伐。坚持绿色低碳发展，大力推进节能减排，城市中心区生活垃圾无害化处理率、资源化利用率分别达到100%和40%。注重生态建设，森林覆盖率达到41.5%，建成区人均公园绿地面积11.3平方米；加强海域海岛岸线整治修复，海域清洁面积达92.7%。强化污染治理，关闭了开采近百年的后盐石灰石矿，整治燃煤锅炉3350台，淘汰黄标车及老旧车辆20多万辆，完成637个村环境连片整治。

　　五年来，我们坚定不移推进开放发展，参与国际合作形成新优势。实施更加主动的开放战略，一批具有里程碑意义的重大开放平台获批，金普新区、跨境电商综合试验区、开放型经济新体制综合试点试验区、自由贸易试验区建设顺利推进，抢占新一轮对外开放制高点。金普新区体制改革深入推进，开放功能进一步凸显。厚植开放优势，累计利用外资365.6亿美元、引进省外内资3696.7亿元，新增世界500强企业投资项目40个；累计进出口总额2989亿美元，占全省的60%、占东北三省的42%。成功承办3届夏季达沃斯论坛，大连以更加开放的姿态走向世界。

　　五年来，我们坚定不移推进共享发展，民生社会事业实现新跨越。践行以人民为中心的发展思想，财政用于民生支出3379.8亿元。累计完成“暖房子”改造1223万平方米、惠及19.3万户，棚户区改造5.3万套、提供公租房保障6万户，增建停车泊位40.9万个。城乡基本养老保险和基本医疗保险制度实现全覆盖，企业退休人员养老金实现十三连增，城乡低保标准居东北首位。基础教育综合水平进入全国前列，医疗卫生体制改革全面深化，社会医疗服务能力大幅增强。社会治理“大连经验”在全国推广，全市生产安全事故起数和死亡人数分别下降82.2%和13.6%。城乡居民人均可支配收入年均增长7.6%和8.6%，均快于GDP增速，城镇登记失业率始终控制在3%以内，人民群众获得感幸福感明显增强。在中国社会科学院2017年《中国城市基本公共服务力评价》中，我市排名上升至第三位。

　　各位代表，同欲者胜，民齐者强。五年来，大连发生的深刻变革、取得的发展成就来之不易，是党中央、国务院和省委、省政府及市委坚强领导、大力支持的结果，是全市广大干部群众砥砺奋进、共同奋斗的结果。在此，向给予本届政府大力支持的人大代表和政协委员，向各民主党派、工商联和无党派人士，向离退休老同志，向中省直各部门和兄弟省市驻连机构，向驻连部队和武警官兵，向关心支持大连振兴发展的海内外朋友们，表示衷心感谢！

　　在总结成绩的同时，我们也清醒看到存在的问题：一是思想观念不够解放，计划经济思维、传统路径依赖等问题不同程度存在。二是体制机制深层次矛盾尚未解决，一些改革部署和政策措施还没有完全落地见效。三是发展质量和效益有待提高，经济结构还需加快调整，产业转型升级任重道远，县域经济实力还不够强，金融和政府性债务风险较大。四是企业技术创新能力不足，全社会科研投入比重偏低，科技与经济融合不够。五是民生领域还存在短板，交通拥堵、教育资源不均衡、优质医疗资源不足等问题亟待解决。六是营商环境仍需大力改善，少数干部不作为、不担当现象仍然存在，一些领域不正之风和腐败问题不容忽视。我们要切实增强忧患意识，高度重视并大力加以解决。

　　三、今后五年的目标任务

　　今后五年，是“两个一百年”奋斗目标的历史交汇期，也是大连率先实现全面振兴、加快“两先区”建设的关键期。今后五年政府工作的指导思想是：全面贯彻党的十九大精神，以习近平新时代中国特色社会主义思想为指导，坚持新发展理念和稳中求进工作总基调，按照高质量发展的根本要求，统筹推进“五位一体”总体布局和协调推进“四个全面”战略布局，持之以恒落实“四个着力”“三个推进”，坚持以供给侧结构性改革为主线，按照市委十二届五次全会的决策部署，扎实做好稳增长、促改革、调结构、惠民生、防风险各项工作，在新时代决胜全面建成小康社会和社会主义现代化建设新征程上奋力走在前列。

　　今后五年经济社会发展的总体目标是：经济步入高质量发展阶段，产业结构持续优化，新旧动能加速转换，全要素生产率明显提升，建成具有大连特点的现代化经济体系。科技创新体系更加完善，自主创新能力显著增强，科技进步贡献率大幅提升，区域性科技创新中心基本形成。重要领域和关键环节改革实现新突破，市场配置资源的决定性作用充分发挥，开放型经济新体制基本确立，参与国际经济合作与竞争优势进一步凸显。贯彻绿色发展理念的自觉性和主动性显著增强，形成节约资源和保护环境的空间格局、产业结构、生产方式和生活方式，主要污染物排放总量大幅减少，生态环境质量总体改善，人与自然和谐共生。城乡居民生活质量全面提升，收入与经济增长保持同步，社会保障和公共服务体系更加健全，社会治理更加有效，人民安居乐业、社会和谐稳定的大好局面更加巩固。“两先区”建设取得重大进展,“十三五”规划的目标任务基本完成，高水平全面建成小康社会，开启大连现代化建设新征程。

　　面对新征程、新目标，我们要振奋精神、坚定信心，综合施策、奋勇争先。

　　一是深入解放思想，在更新思想观念上走在前列。打开思想解放“总开关”，以思想的大解放引领经济社会的大发展。坚持不懈用习近平新时代中国特色社会主义思想武装头脑、指导实践，科学绘制新时代大连发展美好蓝图。全面对标上海，坚决摆脱惯性思维的羁绊、冲破陈旧观念的束缚，以改革开放创新为动力源泉，以前瞻思维、全球视野、国际标准开创大连振兴发展新局面。

　　二是聚力经济发展，在提升质量效益上走在前列。把推动高质量发展作为当前和今后一个时期确定思路、制定政策的根本要求，坚持质量第一、效益优先，以供给侧结构性改革为主线，推动经济发展质量变革、效率变革、动力变革，提高全要素生产率。加大经济结构调整力度，推动传统产业转型升级，培育壮大战略性新兴产业，大力发展现代服务业，提升现代农业质量和水平，加快构建现代产业体系。

　　三是强化创新驱动，在加快动能转换上走在前列。把握世界新一轮科技革命和产业变革大趋势，以国家自主创新示范区建设为载体，加快推进东北亚科技创新创业创投中心建设。着力提升科技创新能力，努力在关键共性技术、前沿引领技术、现代工程技术创新等方面取得突破，逐步在更多领域实现由“跟跑”向“并跑”“领跑”转变。创新人才政策，加强知识产权保护，加快科技成果向现实生产力转化，加快形成以创新引领和支撑高质量发展的新模式。

　　四是推进改革攻坚，在增强发展活力上走在前列。勇立改革潮头，以完善产权制度和要素市场化配置为重点，全面深化经济体制改革。围绕打造国际化营商环境，深化“放管服”改革，提高行政效率，优化政府服务。围绕使市场在资源配置中起决定性作用和更好发挥政府作用，深化国资国企、金融、财税、投融资、农村集体产权等重点领域改革。围绕让人民群众共享改革红利，深化教育、养老、医疗卫生、安全生产等公共服务领域改革。要增强改革的系统性、协同性，提升改革的“联动效益”和“共生效应”，全面激发市场活力和社会创造力。

　　五是提升开放能级，在形成全面开放新格局上走在前列。积极适应经济全球化进程的深刻变化，着力打造更高水平开放型经济。充分发挥自贸区示范引领作用，全面提升贸易和投资自由化便利化水平，培育国际竞争合作新优势。发挥区位优势，加强与“一带一路”沿线国家经济合作。深化开放先导区的体制机制改革，突出对外开放功能，发挥带动效应。积极推进辽宁沿海经济带的开发开放，在协调推进、联动发展上取得突破。加快构建面向全球的贸易、投资、生产、服务网络，进一步增强对外开放门户的聚集辐射能力。加快推进国际航运中心、国际物流中心、国际贸易中心、区域性金融中心和现代产业聚集区建设，增强开放功能，提升城市国际化水平。

　　六是突出绿色发展，在加强生态文明建设上走在前列。树立“绿水青山就是金山银山”的理念，把生态文明建设贯穿于经济社会发展各方面和全过程，全力推进“美丽大连”建设。建立严格的生态环境保护制度，持续实施生态系统保护和修复工程，修复海岸线、河流等自然资源。构建市场导向的绿色技术创新体系，优化能源结构，倡导简约适度、绿色低碳的生活方式。打好污染防治攻坚战，持续改善生态环境，让大连天更蓝、山更绿、水更清、城市更美丽。

　　七是坚持人民至上，在满足人民美好生活需要上走在前列。紧扣我国社会主要矛盾的新变化，把人民对美好生活的需要作为奋斗目标。办好人民满意教育，实施就业优先战略，深化“健康大连”建设。织密扎牢全覆盖、保基本、多层次、可持续的社会保障网，严格落实社会保障待遇政策，不断提高保障能力。推进基本公共服务均等化，全面提升社会治理社会化、法治化、智能化、专业化水平，使人民群众的获得感幸福感安全感更加充实、更有保障、更可持续。

　　四、2018年重点工作安排

　　2018年是全面贯彻落实党的十九大精神的开局之年，是改革开放40周年，是决胜全面建成小康社会、实施“十三五”规划承上启下的关键一年。做好今年的工作，实现新一届政府开好局、起好步，关系长远、意义重大。

　　全市经济社会发展主要预期目标是：地区生产总值增长7%左右，固定资产投资增长10%以上，社会消费品零售总额增长7.5%，规模以上工业增加值增长8%以上，外贸出口增长5%，一般公共预算收入增长7%，单位GDP能耗下降3.5%，城乡居民人均可支配收入与经济增长同步，居民消费价格指数和城镇登记失业率均控制在3%以内。

　　要重点抓好以下几个方面工作：

　　（一）着力推进经济持续平稳增长。

　　大力发展实体经济。着眼建设现代化经济体系，着力推动实体经济健康发展。把发展实体经济切入点放在降低成本和要素供给上，解决企业发展用人、用地、融资等要素保障。把发展实体经济主攻方向放在制造业上，以智能制造为主线推动制造业创新发展，以新一代信息技术为手段推动传统产业转型升级，以引入优质项目为重点拉长产业链，抢占先进制造业制高点。

　　积极扩大有效投资。启动一批重大产业、基础设施、乡村振兴项目建设，跟踪抓好已签约项目落地开工，全年计划完成固定资产投资1820亿元。加大招商引资力度，全面推行企业投资项目承诺制，实际利用外资增长6%以上，引进省外内资1000亿元以上。

　　促进消费加快发展。提升消费层级，优化城市商圈和特色街区，增强消费服务能力。完善鼓励政策，支持社会力量发展教育、文化、养老、医疗、健康等消费性产业。大力发展旅游消费，推进中国邮轮旅游发展试验区建设。推进电子商务发展，打造国际品牌集聚区、时尚消费区。积极引导信息消费，规范网上购物服务。强化消费维权，营造安全放心消费环境。

　　推动外贸持续增长。拓展外贸空间，巩固日韩、欧美市场，全力开拓“一带一路”沿线和金砖国家市场。优化贸易结构，在推动一般贸易发展的基础上，大力发展服务贸易，提高产品和服务的技术含量、附加值和品牌影响力。创新外贸模式，推进跨境电商“六体系两平台”、大型O2O体验店及海运直购业务发展，提高供应链供应水平。强化贸易服务，实施百强外贸企业跟踪服务制度，推进外贸企业孵化基地建设，扶持中小外贸企业发展壮大。

　　支持民营经济发展。放宽民间资本准入门槛，鼓励和引导国有企业与民营企业建立配套协作机制，发展混合所有制经济。积极引导民营企业创新转型发展，重点培育“专精特新”中小企业。推进中小企业公共服务平台网络建设，着力解决中小微企业发展中的困难，支持企业直接融资，着力营造有利于民营经济发展的社会环境。

　　（二）全面提升发展质量和效益。

　　深化要素市场化配置改革。引导企业消除无效供给、增加有效供给，在“破”“立”“降”上下功夫。把去产能与结构调整、产业转型、企业重组、需求管理结合起来，促进供给侧结构性改革由做“减法”为主向做“加法”为主转变。争取投贷联动政策试点，支持企业通过上市、挂牌、债转股等方式降低杠杆率。继续以减税降费为抓手降低实体经济成本。建立多主体供应、多渠道保障、租购并举的住房制度，促进房地产业健康发展。

培育壮大新兴产业。深入实施战略性新兴产业培育和发展三年行动计划，科学布局人工智能、生命健康、洁净能源等未来产业。做大做强集成电路、储能装备两大新兴产业，推进英特尔、融科储能、新源动力、松下电池等项目实现规模扩张和技术突破。持续推动新一代信息技术、生物医药、新能源汽车、新材料、节能环保等优势产业健康发展，促进软件和信息技术服务业向高端化发展，加快打造国内领先的新兴产业集群。

　　优化提升传统产业。积极创建“中国制造2025”国家级示范区，推动信息技术与传统产业深度融合，在中高端消费、创新引领、绿色低碳、现代供应链等领域培育形成新增长点，打造制造业强市。加快金普新区智能装备产业基地、长兴岛石化产业基地建设，确保光洋智能制造、恒力炼化一体化等项目年内建成投产。全力推进中英（大连）先进制造产业园建设。积极创建国家军民融合创新示范区。

　　加快发展现代服务业。以“四个中心”建设为载体，推动生产性服务业向高端化、生活性服务业向品质化发展。以辽宁港口资源整合为契机，加快国际航运中心建设，拓展国际航运服务功能，发展国际中转集拼业务，增强聚集要素能力。加快物流中心建设，推进物流信息化、标准化，完善物流网络布局，推进多式联运发展。加快贸易中心建设，支持大宗商品交易、转口贸易、跨境电商等贸易业态和商业模式集聚发展。加快金融中心建设，完善金融体系，创新金融产品和服务，提高金融服务实体经济效率，突出发展期货、保险、基金、创投等特色金融，打造金融核心功能区。 　　　　　　积极发展海洋经济。建设海洋中心城市，创建现代海洋经济发展示范区，开发海洋新兴产业，发展海洋服务业。推动高技术船舶、海洋工程装备制造业升级。加快海洋牧场建设，打造“蓝色粮仓”，叫响“大连海鲜”品牌，推动海洋资源大市向海洋经济强市转变。

实施质量强市战略。加强标准体系建设，促进经济发展各领域、各层次标准化，引领经济提质增效升级。加强商标品牌建设，推动形成社会资源和生产要素向优质产品、优秀品牌和优势企业聚集的良性机制，让“大连制造”“大连服务”成为高品质高标准的代名词。开展重点产品质量提升行动，弘扬工匠精神，加强全面质量管理；激发企业家干事创业热情，鼓励企业家专注主业、做强实业，打造国际知名品牌。

　　（三）大力实施乡村振兴战略。

　　促进城乡融合发展。重塑城乡关系，坚持以工补农、以城带乡，把公共基础设施建设的重点放在农村，加快农村道路、宽带、垃圾污水处理、饮水安全等基础设施建设，建好、管好、护好、运营好农村公路，新建通屯油路200公里。建立全民覆盖、普惠共享、城乡一体的基本公共服务体系，推动新型工业化、信息化、城镇化、农业现代化同步发展，加快形成工农互促、城乡互补、全面融合、共同繁荣的新型工农城乡关系。

　　完善农村基本经营制度。积极发展多种形式、适度规模经营，培育新型农业经营主体，大力发展家庭农场、龙头企业、社会化服务组织和农业产业化联合体，积极引导工商资本下乡，组建土地股份合作社30家。农业生产经营规模要坚持因地制宜，宜大则大、宜小则小，不搞一刀切，不搞强迫命令，努力把小农生产引入现代农业发展的轨道。

　　深化农业供给侧结构性改革。坚持质量兴农、绿色兴农，加快农村一二三产业融合发展，构建现代农业产业体系、生产体系、经营体系。坚持工业强县、农业稳县、服务活县、人才兴县，发展壮大县域经济。促进“互联网+现代农业”，不断提高农业创新力、竞争力和全要素生产率，加快实现由增产导向向提质导向转变，加快农业农村现代化进程。

　　坚持人与自然和谐共生。统筹山水林田湖草系统治理，加强农村突出环境问题综合治理，建设3处区域性畜禽废弃物资源化处理中心、100个畜禽养殖场废弃物处理设施。建立市场化多元化生态补偿机制，增加农业生态产品和服务供给，实现百姓富、生态美的统一。

　　传承发展提升农耕文明。加强农村思想道德建设，传承发展农村优秀传统文化，加强农村公共文化建设，开展移风易俗行动，培育文明乡风、良好家风、淳朴民风，不断提高乡村文明程度。

　　创新乡村治理体系。建立健全党委领导、政府负责、社会协同、公众参与、法治保障的现代乡村社会治理体制，健全自治、法治、德治相结合的乡村治理体系，促进乡村善治，确保乡村充满活力、和谐有序。

　　打好脱贫攻坚战。引导128个低收入村发展优势特色、优质高效产业，增强“造血”功能，确保到2020年全市低收入村人均可支配收入达到14000元以上。

　　（四）加快建设创新创业创投中心。

　　加强创新体系建设。深入实施自创区建设三年行动计划，积极推进洁净能源国家实验室、“大连光源”基地建设，推动人工智能、水下机器人、干细胞与组织工程等10个高端产业技术创新联盟和60个创新研发平台建设。发挥国家“双创”示范基地等引领作用，构建众创空间—孵化器—加速器全链条创业孵化体系，支持在连高校建设大学生创新创业基地。实施创新型领军企业、高新技术企业、小微科技企业培育计划，新增高新技术企业200家、初创科技企业3000家。

　　实施科技创新攻关。瞄准世界科技前沿，强化基础研究与应用研究，努力在引领性原创性成果、关键共性技术上实现突破。在人工智能等领域实施10项科技创新专项，在先进装备制造等领域启动一批技术研发项目，为创新发展提供有力支撑。

　　加速科技成果转化。打造科研机构、高校、企业共同参与的科技成果转化平台，建设技术专利交易市场。围绕重点产业领域，建设一批专业性行业技术转移机构，推动国家技术转移东部中心大连分中心建设。实行专利资助奖励政策，启动专利导航项目，万人有效发明专利拥有量达到18件以上。

　　推进科技体制改革。完善支持企业技术创新政策，加大研发费用加计扣除、高新技术企业税收优惠等政策落实力度。深化科技成果使用权、处置权、收益权改革，构建市场导向、知识价值导向的科技成果转化机制。实施科技人才创新创业支持计划，培养科技领军人才和顶尖创新团队。

　　（五）持续深化重点领域改革。

　　深化营商环境建设。放权要更彻底、更到位，巩固“多证合一”成果，推广企业登记全程电子化和电子营业执照应用，实行“证照分离”，实施市场准入负面清单制度。监管要更科学、更高效，实行“六个双”监管方式，推动跨部门“双随机、一公开”监管。服务要更精准、更贴心，出台行政审批标准，推进建设项目并联审批；升级网上审批服务平台，推动“互联网+政务服务”向基层延伸，所有行政审批事项办结时限不超过国家规定时限的40%。

　　深化国企国资改革。优化国有资本布局，扩大国有资本在高端制造、新能源、节能环保等新兴产业中的比重。推进国有企业股权多元化改革，支持国有控股的混合所有制企业实施员工持股。按照现代企业制度要求，完善法人治理结构和灵活高效的运营机制。

　　深化行政体制改革。加强与国家、省行政体制改革衔接，统筹各类机构设置和使用各类编制资源。深化开放先导区体制机制创新，赋予其更多自主权。继续推进事业单位分类改革，按照市委深化改革领导小组的部署，积极稳妥推进4个方面14项改革。

　　深化金融监管体制改革。强化地方金融监管部门职责配置，确保监管责任接得住、管得好。建立各级政府债务化解和应急处置机制，分级落实政府债务偿还责任，严控政府债务增量。整顿规范金融秩序，建立多部门联动机制，坚决守住不发生系统性区域性金融风险底线。

　　（六）积极构建开放型经济新体制。

　　加快自贸试验区建设。把制度创新作为主攻方向，全面完成149项创新复制推广任务，国际贸易“单一窗口”建设要达到上海3.0版水平，着力在投资、贸易、金融、管理、服务等领域形成一批可复制可推广的“大连经验”。坚持自贸区与自创区联动发展，促进科技、金融、投资、贸易、产业多维融合，放大功能集成效应。全力申办自由贸易港。

　　增强先导区对外开放功能。进一步明确各园区功能定位，完善管理体制，强化功能作用。推进基础设施建设，打造各具特色的智慧园区。加大招商引资力度，以项目论英雄，创新激励政策，优化投资环境。建立体现先导区特点的人事管理制度，形成干部使用与工作成效挂钩机制。

　　扎实推进沿海经济带开发开放。落实省委、省政府要求，发挥大连龙头带动作用，促进辽宁沿海六市资源共享、优势互补、错位发展。推进基础设施互联互通、产业发展协同协作、市场要素对接对流、公共服务共建共享，形成生产要素聚集的高地。

　　深度融入“一带一路”建设。推进海外生产基地、研发中心、营销服务中心以及跨境经贸合作园区建设，带动产品、技术、标准、服务“走出去”。开展投资、贸易、金融、人文及基础设施建设等多领域交流合作。

　　全面提升沪连对口合作水平。实施一批标志性跨区域合作项目，推进沪连合作产业园区建设。在体制机制创新、城市功能提升、科创中心建设、人才培养服务等领域取得务实成果。

　　（七）精心打造美丽宜居典范城市。

　　科学编制城市未来发展规划。尊重城市发展规律，坚持“五个统筹”，贯彻“适用、经济、绿色、美观”的方针，着力转变城市发展方式、塑造城市特色风貌、改善城市环境质量，提升城市规划水平。注重城市设计，强化建筑管理，传承历史文脉，建设特色大连。坚持以提升综合创新力、国际竞争力、可持续发展能力和城市独特魅力为重点，明确城市未来的愿景目标、功能定位、发展战略、行动计划和阶段性任务。坚持“三个向北”转移，优化城乡空间格局和产业布局，拉开城市发展框架。

　　完善城市基础设施。加快交通基础设施建设，推进新机场、地铁4号5号线、大连湾海底隧道、金普城际铁路、旅顺中部通道、大连北站综合交通枢纽等工程。提升市政基础设施建设水平，加强停车场建设和智慧化管理，推进充电桩、加气站规划建设；加快推进天然气进城管网建设，完善东港、梭鱼湾、凌水湾、新机场商务区基础设施；加强城市地下综合管廊建设和地下空间开发利用，维修200万平方米老旧住宅下水管网；推进市区公共机构、公共场所生活垃圾强制分类和居民小区垃圾分类试点；实施老旧小区改造，制定物业服务标准和规范。

　　加快智慧城市建设。推进全光纤网络城市建设，实现无线宽带网络全覆盖，推进数据资源共享，构建智慧化城市基础设施运行管理体系。整合资源，加速电子政务云中心建设，打通“信息孤岛”。

　　大力提升文化软实力。弘扬社会主义核心价值观，支持文艺创作，提高文化供给能力和服务质量，推动文化繁荣发展。深化群众性精神文明创建，提升城市文明程度和市民文明素养。加强文物保护和文化遗产传承，抓好旅顺太阳沟、西岗东关街等历史文化街区和冰山、机车等老厂房的保护和合理利用。积极引导文化消费，培育壮大文化产业。

　　推进城市精细化管理。坚持以人为本、依法治理，着力提升城市品质、体现城市之美。全面提升山体公园建设水准，开展行道树补植工程，加大亮化、美化力度，精心营造大气、整洁、有序的市容市貌。深入推进城市环境综合整治，重点解决交通拥堵以及停车难、停车乱等问题，让城市更有序、更安全、更干净，市民生活更方便、更舒心、更美好。

　　加强生态环境建设。完成生态保护红线划定，实施城乡十大造林工程，保护和利用好滨海景观资源，塑造鲜明的海滨城市风貌。着力解决突出环境问题，全面落实“大气十条”“水十条”“土十条”，继续打好蓝天保卫战，加强臭氧防治和扬尘管控；全面推进河长制，实施流域环境和近岸海域综合治理，推进城市生态河建设和海绵城市试点工作；强化污染地块修复。深化中央环保督察和国家海洋督察反馈问题整改，加强海岸、海湾、海岛生态保护和修复，坚决禁止劈山毁林、围海造地等破坏生态环境行为，保护好大连的好山好水好风光。实施领导干部自然资源资产离任审计。

　　（八）坚持在发展中保障和改善民生。

　　坚持教育优先发展。编制教育现代化规划，落实立德树人根本任务。推进义务教育集团化办学，扩增普通高中优质资源，努力办好市民家门口每一所学校，让每个孩子都能享有公平而有质量的教育。扩大普惠性幼儿园覆盖面，新增公办幼儿园15所、学位3000个。支持在连高校与城市融合发展，促进“双一流”建设。支持社会力量办学。推进职业教育资源整合，完善职业教育和培训体系。

　　提高创业就业水平。全面落实职业培训补贴政策，以创业带动就业，健全覆盖城乡的公共就业创业服务体系，统筹推进高校毕业生和农民工等重点群体就业创业。着力增加居民经营性、财产性收入，建立科学合理的企业工资正常增长机制和支付保障机制。

　　健全社会保障体系。按照兜底线、织密网、建机制的方针，构建覆盖全民、城乡统筹、权责清晰、保障适度、可持续的多层次保障体系，不断提升保障服务能力和社保待遇水平。健全社会救助体系，提高城乡低保、特困供养标准。大力复制推广城镇居家养老“林海模式”，今年再推进建设50个社区居家养老服务中心。

　　积极推进健康城市建设。深化医药卫生体制改革，健全现代医院管理制度，加强重大疾病防控。实施改善医疗服务行动，加强基层医疗卫生服务体系和全科医生队伍建设，支持发展中医药事业。持续发展残疾人事业，优化残疾人康复服务。加大国家食品安全示范城市建设力度，让人民吃得放心。广泛开展全民健身活动，支持足球事业发展，举办好国际性体育赛事，建设体育强市。

　　解决好突出民生问题。用心用情确定和实施一批体现托底性、普惠性、导向性、协同性的民生工程，集中力量解决群众高度关注的堵点、痛点、难点问题，切实让群众感受到大连城市的温度。注重慰老、爱幼、扶弱、济困，解决特殊困难群众最迫切的民生难题；增加公共服务供给，重点向农村和薄弱地区倾斜；倡导绿色健康生活，提升市民居住环境和生活品质；增强安全保障能力和城市运行效能。

　　构建有效社会治理体系。进一步完善市民服务管理、矛盾纠纷化解、社会组织孵化、平安大连建设四大体系，实现全体市民共治、共建、共享。加强社会治安综合治理，严厉打击违法犯罪活动。严格落实安全生产责任制，坚决遏制重特大事故发生。强化消防安全管理，提升灭火救援能力。加强信访法治化建设，实施信访矛盾减存控增三年攻坚计划。推进公共法律服务体系建设，为人民群众提供优质高效的公共法律服务。

　　继续做好民族、宗教、外事、侨务、对台、人防、档案、气象、仲裁、防震减灾、双拥共建、国防动员、民兵预备役和边海防等各项工作。

　　（九）建设人民满意的服务型政府。

　　加强各级政府党的建设。坚持管党治党责任与政府工作责任一起落实，切实把全面从严治党要求落实到政府工作全过程。在广大公务员中广泛开展“不忘初心、牢记使命”主题教育，牢固树立“四个意识”，增强“四个自信”，严守政治纪律和政治规矩，提高全体公务员的政治站位，自觉践行习近平新时代中国特色社会主义思想。

　　加快建设法治政府。健全行政决策机制，推进重大行政决策科学化、民主化、法治化。深化执法体制改革，规范行政执法行为，做到公开公正文明。主动接受人大法律监督和工作监督、政协民主监督、社会舆论监督，让权力在阳光下运行。

　　持续推进作风转变。持之以恒正风肃纪，严格落实中央八项规定实施细则精神，深入查找“四风”突出问题特别是形式主义、官僚主义的新表现，以永远在路上的韧劲抓好作风建设。大兴调查研究之风，深入基层，深入群众，查真情、摸实底、出实招、求实效。大兴真抓实干之风，坚持马上就办、办就办好，敢于担当，主动作为，善抓落实，善作善成。

　　全面强化廉洁从政。大力推进党风廉政建设和反腐败斗争，注重源头治理，扎紧制度的笼子，规范权力运行，严格落实“一岗双责”，深入落实《准则》《条例》，减少腐败存量，遏制腐败增量，以廉洁从政的实际成效取信于民，树立政府良好形象。

　　各位代表！善学者尽其理，善行者究其难。建设“两先区”既是习近平总书记对大连的殷切期望，也是全市人民必须为之奋斗的目标。让我们更加紧密地团结在以习近平同志为核心的党中央周围，在市委的坚强领导下，以时不我待、只争朝夕的精神投入工作，锐意进取、埋头苦干，为谱写新时代大连振兴发展的华彩乐章而不懈奋斗！
